# A bridge over sustainable water: Politicians’ perceptions about the preconditions for collective action

**DOI:** 10.1007/s13280-023-01975-5

**Published:** 2024-02-07

**Authors:** Anna Bendz, Patrik Öhberg

**Affiliations:** 1 Department of Political Science, University of Gothenburg, Box 711, 405 30 Göteborg, Sweden; 2 SOM-Institute, University of Gothenburg, Seminariegatan 1B, 413 13 Göteborg, Sweden

**Keywords:** Collective action, Politicians, Survey experiment, Sustainable water management, Sweden, Upstream/downstream-relations

## Abstract

Sustainable water resource management is a core interest for all societies. As water systems are often common resources, the management of water systems requires coordinated action among actors along the water. For flowing water, a complication for coordination is upstream–downstream relations where what happens upstream affects downstream, but not the other way around. In this study we present results from a survey experiment with politicians in Sweden, focusing on whether and to what extent their willingness to cooperate is affected by their placement upstream or downstream along a fictive water system. Our findings indicate that politicians from upstream and downstream municipalities share the view that upstream politicians bear greater responsibility for undertaking preventive actions and are willing to assume remedial responsibility for problems caused by them. These results challenge the notion that self-interest is the primary obstacle to resolving environmental collective action problems.

**Supplementary Information:**

The online version contains supplementary material available at 10.1007/s13280-023-01975-5.

## Introduction

The achievement of a sustainable water resource management in order to safeguard water quality is a core interest for all societies. A broad range of climate related incidents have been identified as factors affecting the quality of water, including e.g., rising sea levels, heavy rain, and flooding. To ensure future access to clean water, it is necessary to carefully manage present and future risks and prevent serious events that threaten the wellbeing of humanity and the functioning of society.

Since water systems often are common resources and most risks to water quality are transboundary in character (see e.g. Linnerooth-Bayer et al. 2001; Tait and Bruce 2001; Renn 2008), cooperation and coordination among and across administrative units is necessary, which is also clearly declared through numerous politically binding documents, e.g., the EU Water Framework Directive (Directive 2000/60/EC). The realization of this goal is, however, often challenged by diverging and sometimes strong interests among decision makers. Even though several actors on different levels are typically involved in water resource management, the responsibility for providing citizens with clean water is usually a decentralized task with local governments as the responsible government level (Bendz and Boholm 2019), and it is thus local politicians that ultimately have the power to decide on whether to collaborate with other actors in order to promote the common good; even if that would mean that they need to refrain from their own and/or their constituents’ short-term interests. As such, local politicians are key actors in solving the challenges of water-related cooperation and their political incentives and representative tasks need to be taken into consideration. Although collaboration between local stakeholders is seen as important in the case of solving water management problems, few studies focus on local governments and what drives them to participate in collaboration or not (Hoornbeek et al. 2016; Yoder et al. 2021).

For flowing water, the upstream–downstream relations in watersheds, i.e., where the use of water upstream affects people living downstream but not the reverse (Savenije and van der Zaag 2008), complicates the possibilities for coordination of efforts to achieve sustainable water management. Upstream users need to consider the consequences for downstream users when planning how the water and their surrounding land should be used. Thus, scholars have identified the upstream–downstream relations along water systems as a particularly difficult problem to handle when it comes to the management of transboundary water systems (Le Marquand 1977; Lundqvist and Falkenmark 2000; Lubell et al. 2002; Lubell and Balazs 2018).

The management of a running water system is a schoolbook example of a collective action problem where upstream actors need to act (coordinate their actions) for the interest of downstream actors to be ensured. Yet, problems concerning the coordination of streaming drinking water risk management can be understood as collective action problems with *asymmetric payoff*s. Cooperation and coordination among one or more upstream actors is necessary for reaching collectively beneficial outcomes, but where the negative consequences of defecting from that cooperative behaviour will mainly or solely have negative consequences among downstream actors. Thus, it is rational for downstream actors to cooperate by, e.g., supporting initiatives aimed for risk prevention upstream. However, it is less apparent what would motivate upstream actors to act collectively by accepting to take costs that are not particularly beneficial for themselves.

In this article, we study politicians’ propensity to accept upstream and downstream collaboration aimed at safeguarding water quality, from their positions as representatives for either an upstream or a downstream municipality. We aim to analyze what remedial responsibility positions politicians adhere to when attributing upstream- and downstream actors with responsibility for managing problems in the shared water. We investigate this by conducting a survey experiment involving politicians in Sweden, where the responsibility of providing citizens with clean water rests with the local municipalities.

## Theoretical framework

In most countries, drinking water supplies are local natural resources, and the water is treated and distributed locally. Drinking water risk management is therefore paradigmatically a responsibility of local governments, even though national and regional levels are also typically involved in regulation, planning, supervision, and law enforcement. Since neither streaming water, nor global warming and climate change respect any administrative borders, planning and cooperation across municipal jurisdictions is necessary but not necessarily compulsory (Lundqvist 2016). According to the EU Water Framework Directive, introduced by the European Union in 2000, freshwater resource management should no longer be organized by existing administrative hierarchies, but rather by the natural logic of the water catchment areas transcending local, regional, and even national boundaries (Directive 2000/60/EC). The risk of deteriorating water quality is intricately linked to future climate change and various transboundary environmental hazards. These factors cannot be adequately managed within a single municipality’s decision-making structure. Our contention is that collaboration among municipalities using a common water system is necessary in order to effectively cope with the risks at hand (Leroux and Carr 2007; Feiock 2013). However, inter-municipal collaboration sometimes requires that local decision makers are willing to put aside their own municipality’s immediate self-interest to achieve what is best for the larger collective (Feiock 2009, 2013).

The problem concerning how drinking water risk management should be coordinated among local decision makers can be understood as a collective action problem: unless the involved actors choose to cooperate in managing the joint water source, there will be negative outcomes for the collective as a whole. A collective action problem is normally described as a social dilemma situation (Dawes 1980) where actors’ individual short-term self-interest conflicts with longer-term collective interests. This is a situation which can generate a substantial risk that the collective benefit is not produced at all. Famously, Olson (1965) made the presupposition that rational and self-interested actors will typically not opt for collective action if the benefits of such actions do not clearly exceed the individual cost. This means that the decision-makers and residents of municipalities can be expected to oppose efforts to inter-municipal cooperation on clean water if they perceive that the costs for them exceed the perceived benefits. This situation is very much present in the case of flowing water since the benefits of upstream endeavors mainly constitute itself further down the river. And vice versa; down-stream users are typically more negatively affected by incautious behavior committed further up the stream. Constitutive for collective action problems are that they cannot be managed or vanquished unless at least some actors act against their first-order preferences, i.e., their short-term self-interest or the prime interest of their principals, thus adopting a cooperative rather than a defective behavior.

The management of a running water system is a clear case of a collective action problem where upstream actors need to act, or be coordinated, for the interest of downstream actors to be catered for. More specifically, the challenge of coordinating streaming (drinking) water is a collective action-problems with *asymmetric payoffs* and successful cooperation and coordination implies the need for different kinds of responsibility-taking among the involved actors to reach collectively beneficial outcomes. The consequences of a defecting behavior upstream, will only (or at least mainly) have negative consequences among downstream actors, while cooperative behavior executed down the stream is not automatically (or at all) beneficial for the collective good per se. This feature makes upstream- versus downstream responsibility- a rather intriguing case of collective action (e.g., Ostrom and Gardner 1993; Swallow et al 2006; Cardenas et al 2010).

Several studies demonstrate that upstream–downstream relations are challenging and that they make collaboration on watersheds complicated because of its asymmetrical nature and lack of interdependence (Sangkapitux et al 2009; Pfaff et al 2019; Yoder et al. 2021). Collaboration between different kinds of actors on different levels has been put forward as a means to tackle such complex water management problems worldwide in order to enable participation in environmental decision making that leads to a beneficial outcome (Margerum and Robinson 2015). Here, locally based institutions have been demonstrated to be of importance to reconcile and monitor upstream/downstream interests. (Pfaff et al 2019). The decisions of local governments are, in most contexts, vital in order for collaboration on the local level to result in a beneficial outcome. However, as results from an interview study with Swedish local decision makers demonstrate, even though local decision makers recognize the need for collaboration, power and resources imbalances as well as self-interest and distrust may prevent participation (Bendz and Boholm 2019).

In deciding whether to engage in collaboration or not, local decision makers need to deal with several considerations. From a rational vote-maximizing perspective, decision makers should consider how collaboration would affect their possibilities to remain in power. Public opinion is thus part of the calculation when decision makers decide if they should promote collaboration or not (Hood et al 2001). To achieve legitimacy for political decisions that intend to promote the interests of a larger collective, it is necessary that the citizens of the municipalities support, or at least are not opposed to, the decision.

There is ample evidence that public opinion both constrain (Sobel 2001; Foyle 2004) and direct the actions of decision makers (Soroka and Wlezien 2010). If public attitudes are negative, decision makers have less incentive, both from a democratic-theory (i.e., acting as the voice of the people) and a vote-maximizing perspective to engage in collaborative arrangements. At the same time, the management of running water is seldom a salient issue among voters and is likely perceived as a technical problem that should be handled by experts. A study on local decision makers in the municipalities surrounding the Göta Älv water system in Sweden revealed that they perceive citizens as unengaged in drinking water issues in general, taking unlimited access to clean drinking water for granted, and have very limited knowledge concerning the different risks that threatens this access (Bendz and Boholm 2020). This implies that citizen attitudes are less of a constraint for local politicians when it comes to water management.

Moreover, politicians are seldom rewarded by voters for taking preventive action. For example, research related to natural disasters has shown that voters are more likely to support politicians who deliver disaster relief after a disaster as opposed to disaster prevention (Healy and Malhotra 2009). Therefore, politicians might not choose to invest in disaster preparedness, which evidence also suggests. It might be better for politicians to give disaster relief since it is more rewarding and give their voters immediate and visible help. Yet, natural disaster is obviously nothing politicians wish for. Relief is likely to be more expensive than prevention. Policy-oriented politicians may have concerns that allocating resources to disaster relief efforts could potentially hinder their ability to implement investments in other areas. However, recent research indicates that parties addressing flooding issues are likely to gain popularity among the electorate (Birch 2023). Consequently, politicians’ evaluation of their responsibility in relations to collaboration of water systems is an intriguing issue.

In terms of politicians’ understanding of their responsibility, decision makers can, first, be ascribed *causal responsibility* for the problem. Decision makers can cause a problem for which they are responsible, e.g., by making decisions leading to polluting substances are emitted into the water by not allocating money to repair infrastructure or by demanding products being produced in a manufacturing process that emits polluting substances into the water etcetera. A second important aspect concerns who ought to take responsibility for *amending* a problem. One potential answer is to share burdens equally between the involved actors. In many instances of environmental politics, the prevailing solution is often based on the Polluter Pays Principle (PPP). PPP is well-established and stating that the actor(s) responsible for causing the problem also simultaneously and immediately has *remedial responsibility* to amend it: “To be remedially responsible for a bad situation means to have a special obligation to put the bad situation right, in other words to be picked out, either individually or along with others, as having a responsibility towards the deprived or suffering party that is not shared equally among all agents.” (Miller 2001, p. 454.)

Remedial responsibility relates to the issue of how to share (climate) burdens, where the concept of compensatory burdens refers to the recompense of agents for being affected by undeserved aversive events or risks. The question is who should compensate (Page 2011). In the literature of ethical and political-theoretical literature concerned with climate change adaptation, several additional principles other than PPP have been identified as potential reasons for ascribing remedial responsibility to an actor, including historical emissions or the ability to pay (where the agents with the most capacity should shoulder the burdens) for remedial operations (Page 2011). An actor could thus be ascribed remedial responsibility for a problem even though not (currently) causing it. In the discussion, the distinction between currently causing and historically having caused a problem is often problematic since climate change is a function of cumulative CO_2_ emissions. In the case of polluting running water, the cumulative aspect is less prominent since pollution often has an immediate effect that can be attributed to a specific time-bound event.

In the case of upstream–downstream water management, the questions of causal and remedial responsibility may be of significant relevance when it comes to motivating actors to act collectively. Upstream actors typically cause the bad situation downstream—as such being causally responsible as well as potentially activating a sense of remedial responsibility resembling PPP. For upstream actors, it is a matter of whether they are willing to take causal responsibility for events that occur upstream, even if they are not affected—thereby accepting also remedial responsibility for amending or preventing the problems. For downstream actors, it is a question of how willing they are to take on responsibility for sharing the burdens even in situations where they have not caused the problems. For downstream actors, it could make sense to compensate upstream actors for taking necessary remedial action, with references to principles such as ability to pay or that they are the beneficiaries of upstream precautions (e.g. Caney 2001; Page 2011).

Both groups of actors can choose to defect by choosing to act in their (constituents’) self-interest (see further below) instead of cooperating. Choosing not to cooperate risks aggravating a sustainable water management, as it could result in neither upstream nor downstream actors will be willing to contribute to risk preventing actions.

In the watershed collective action situation, it is possible to envision at least four different responsibility positions, each of which may generate different opinions among upstream- and downstream decision makers.

### Self-interest

From a collective action perspective, the perhaps most straightforward theoretical assumption concerns that actors will act in self-interest (Olson 1965) This means, first, that upstream actors will not accept responsibility for causing harm downstream. Second, from this assumption follows that it will be most rational for downstream politicians to hope that upstream colleagues will take responsibility. This means that downstream decision makers will insist on letting upstream actors bear the costs for risk prevention and incident management, while not having to increase their own fees for water services. However, the best possible outcome for upstream politicians is of course to not take on responsibility and do nothing to prevent risks, since that will leave them with the highest positive pay-off.

### Causal responsibility

Due to the constitutional situation in Sweden, for upstream municipalities (and their local actors), any watershed collective action must be voluntary and internally motivated, based on a sense of solidarity with those living further down the river. As downstream is vulnerable to upstream pollution and not the other way around, downstream politicians can only hope for upstream municipalities to admit causal responsibility and accept remedial responsibility. Such “hydrosolidarity” has previously been suggested to facilitate in solving larger scale upstream/downstream conflicts. In a broad sense, hydrosolidarity refers to a kind of ethics around water management of shared water resources, including propensity to cooperate and attention to the common good (Falkenmark 1999; Gerlak and Varady 2009; Gerlak et al 2011). Of particular importance is that upstream decision makers recognize that their actions influence people downstream (Falkenmark 2001). Aspects of hydrosolidarity has been applied in several river basin cases, for example by influencing institutional arrangements and encouraging agreements between stakeholders (e g Pigram 1999).

### Conditional altruism

An important hinder for the realization of hydrosolidarity, put forward by critical scholars, is the lack of incentives for upstream politicians to give up their self-interest for the sake of downstream users. Even though solidarity or good neighbourliness can be of importance in upstream/downstream relations, upstream politicians cannot self-evidently be expected to agree on acting solidary without material compensation and knowledge about the problems and risks they are causing downstream. Thus, first, it might be necessary to increase incentives to politicians upstream so it would be less costly to act against their self-interest (Gerlak and Varady 2009). van der Zaag (2007) suggests that the way to balance the asymmetrical relation between upstream and downstream is by a reversed ‘flux’ in the direction of upstream, either by compensating with material resources or a plead for solidarity. Actors may be conditional cooperators forming the opinion that (a) I ought to give you some compensation, if and only if, you take responsibilities that benefit me. And vice versa, of course, (b) I am willing to take responsibility if and only if I become sufficiently compensated for my sacrifices.

### Equal burden sharing

A final motivation for a decision maker to take on a financial or other burden may be if he or she is guided by the idea that burdens should be shared brotherly. In the situation with the watershed upstream downstream collective action problem, this principle should probably be more appealing for downstream politicians, since it implies that only half of the burdens will have ascribed them, while the rest will be allocated to actors further upstream. In the same way, this motivation is probably less attractive to upstream politicians, since the obvious alternative is that they do nothing to improve the situation for those actors located further down the stream.

It is important to note that it is fully possible that both upstream- and downstream decision makers may form both positive and negative opinions about several of the responsibility positions. Thus, in the following empirical analyses, we do not hypothesise that the upstream- and downstream decision makers will *only* form, e.g., a positive opinion about one of the responsibility positions and will be negatively oriented towards all others. Rather, we are empirically interested in seeing *if* there is variation between politicians’ opinion about different remedial responsibility positions, and if these differences are related to whether the respondents are upstream- or downstream residents. According to our theoretical exercise above, such differences should be expected.

Based on this, we ask the following questions:

1. What opinions concerning different responsibility positions are expressed by the politicians?
2. Can potential differences in opinions be attributed to where decision makers are located along a streaming watershed?

## Drinking water management in Sweden

We use Sweden as a case study, a country where clean water has been an abundant resource for a long time, but where increasing threats from foremost climate change to the quality of tap water, and more frequent incidents involving drinking water, has caused the authorities to point out the importance of taking measures to secure access to drinking water also in the future.

Since the 1860’s, when the first public waterworks was established in the capital of Stockholm, clean and safe drinking water has been taken as more or less self-evident in Sweden. However, today water quality is, as mentioned above, increasingly threatened, both by the repercussions of climate change and other factors. (Boholm and Prutzer 2017). Consequently, in a series of investigations by the Swedish government, drinking water has been identified as a critical resource at risk from future effects by climate change (SOU 2006, p. 196; 2014, p. 53; 2015, p. 51; 2016, p. 32). Some parts of Sweden, for example, risk lower levels of ground water and thereby a possible deficit of drinking water, something that in fact is already regularly the case. The list of possible risks for safe drinking water can obviously be made long, apart from those related to climate change, risks also origins from for example poor infrastructure, which has caused an increased need for heavy investments, or pollution from agriculture or industries (Bendz and Boholm 2019).

Although Sweden is a case where substantial changes have been made in relation to the EU Water framework directive (WFD), responsibility for water management is still strongly connected to the local muncipalities. The local land-use planning mandate, referred to as “local planning monopoly”, as well as a right to self-government makes the locally elected politicians important actors in water governance (Hedelin et al. 2023).

Sweden has 290 municipalities, which have a high level of local self-government. Within boundaries of national legislation, they can make priorities and choices regarding the provisioning of services to citizens. Municipalities are responsible for supervision and enforcement of regulation, and service provisioning within several areas within their geographical jurisdiction, among them drinking water provisioning and waste-water management.^1^ Municipalities are legally required to provide drinking water and to manage service production including waterworks, pipelines, and other facilities and responsible for identifying and managing risks. In contrast to other areas of municipal responsibility, drinking water and waste-water management are funded by fees rather than taxes. The fee is based on the costs for the service divided within the collective of users and decided by politicians in the municipal city council. Most Swedish citizens get their water from publicly administered water systems. The principle of self-government means that local decision makers are key actors when it comes to drinking water risk management. It is the local government that decides whether to collaborate with other municipalities, and in that case how.

Due to the complex and fragmentary regulatory framework for water management, there are quite a substantial number of actors involved in the process of guaranteeing good drinking water to the citizens. Apart from municipalities, national, regional and local authorities as well as different kinds of organisations and stakeholder groups engage in interconnected and partly overlapping responsibilities within the complex risk governance network of regulatory bodies, stakeholders, public and private actors (Boholm et al. 2012; Boholm and Prutzer 2017; Karlsson 2010; see also Lewis et al. 2013).^2^

## Materials and methods

We address our questions through an experimental design on data from the Panel of Politicians, run by the SOM Institute at the University of Gothenburg.^3^ The Panel of Politicians is an online survey and consists to date of 3000 politicians from national, regional, and local levels (for previous work, see e.g., Öhberg and Naurin 2016; Butler et al. 2017; Esaiasson and Öhberg 2020) and has been running since 2011. The panel is proportionally represented in the sample, except for the anti-immigrant party, Sweden Democrats. For the representativeness of the panel compared to the population of Swedish politicians, see fig S5–7, Supplementary information. The data was collected from the beginning of June to the end of July 2022. The response rate for the panel was approximately 40 per cent. One of the experiment groups (*n* = 591) were asked to imagine themselves living upstream whereas the other experiment group (*n* = 610) conversely were asked to imagine themselves living downstream. The respondents in the two experiment groups are hereby referred to as upstream and downstream respondents respectively (see Table S1, Supplementary information for representativeness and random checks). It is a rather common practice to use vignettes when asking politicians on their potential actions in various situations, see for example: Butler and Dynes (2016), Wouters and Walgrave (2017), and Sheffer et al. (2023)

In the experiment, all respondents were first provided with the following introductory text (translated from Swedish):

In Sweden, we have access to a lot of water, but clean water is not self-evident. It takes measures to secure the water quality. Today, there are several threats to clean water. About half of the country’s water systems do not fulfil the demands of ‘good water-status’ which is a measure of the ecological and chemical water quality. In society, there is also substances that are hard to break down and that can get into our waters, which puts high demands on purification of the water.

Climate change is expected to increase precipitation and increase the risk of pollution in waters used as drinking water sources. Many of the country’s drinking water sources lacks a good enough protection against increased pollution. In order to secure the access to clean water also in the future, there is a need for substantial investments.

After the introduction a scenario was presented, completed with a map, showing a fictive water course and where crosses and arrows marked locations upstream and downstream. The scenario read as follows for upstream residents, with alternative text presented to downstream residents in [brackets]. The replaced wordings in the upstream scenario are here shown in **bold**:

Imagine that you are a politician in one of several municipalities located **upstream** [downstream] along a water course, used as a drinking water source. Because of a lack of maintenance of pipelines **in your municipality** [a municipality upstream], an emission of bacteria is let out in the water (see the black cross on the map). The consequence is that citizens **in several municipalities downstream (see the direction of the arrow on the map)** [in your municipality] are afflicted by severe health problems and have to boil their drinking water for a longer period.

Following the scenario-text, questions were posed to capture attitudes to cooperation between upstream and downstream municipalities, here used as dependent variables.

We captured preferences for cost distribution by the question: How do you think that the costs for decreasing the risks that threatens the drinking water should be distributed between citizens in municipalities upstream (where water runs from) and citizens in municipalities downstream (where water runs to) the river? A seven-point scale was employed, ranging from’citizens upstream should pay the full cost’ (1) to’citizens downstream should pay the full cost’ (7) with’equal share’ as a middle alternative (4).

In the survey, a battery of questions about cooperation between municipalities were included. The questions were constructed as statements, which the respondents were asked to take a stand to by choosing from a scale with the following alternatives: totally agree, partly agree, hardly agree, do not agree at all (the complete response rates for each item are presented in the appendix).

1. Municipalities upstream are responsible to make investments that reduce the risk for pollution of the water that can affect downstream municipalities.
2. Municipalities downstream should contribute with money to municipalities upstream in order to help out with costs for measures that prevent pollution of the water.
3. If the water is polluted because of something occurring upstream, municipalities upstream should compensate downstream municipalities for the costs.
4. Every single municipality is responsible for making investments in order to prevent events that pollute the water, irrespective of the consequences do not affect the municipality’s citizens.

Table 1 below shows how we connect our four remedial responsibility positions to the questions and statements above. The scenario concerns an event that has occurred and caused severe consequences downstream, and the questions refer to preventive actions. Thus, they measure the willingness to take action to make sure the water does not get polluted in the first place. This means that decision makers may need to invest in infrastructure or other costly measures, which in turn could make it necessary to raise the water fees in their municipality. By designing the experiments in this way, we make it clear to the respondents that taking responsibility could be costly, and that there is a choice between contributing to the common good and local self-interest (to keep costs down). For local politicians, raising fees or prioritize using tax revenues for investments in preventive measures that mainly benefits people in other municipalities, could be potentially risky as it may cost them votes in the next local election.

**Table 1:** Remedial responsibility positions and the survey items used to measure them

Remedial responsibility position	Survey items
Self-interest	Distribution of costs between upstream and downstream municipalities
Equal contribution	Every single municipality is responsible for making investments to prevent events that pollute the water, irrespective of the consequences do not affect the municipality’s citizens. (+)
Causal responsibility	Municipalities upstream are responsible to make investments that reduces the risk for pollution of the water that can affect downstream municipalities (+)
Conditional altruism	Municipalities downstream should contribute with money to municipalities upstream in order to help out with costs for measures that prevent pollution of the water (+) If the water is polluted because of something occurring upstream, municipalities upstream should compensate downstream municipalities for the costs (+)

In drawing conclusions concerning self-interest, we assume that the short-term self-interest of both the upstream and downstream respondents would *not* support remedial responsibility for their own location, but instead insist on letting the other group bear the costs for risk prevention, and thus not having to increase their own fees for water service or use other parts of the municipal budget to make investments. The other three positions are measured by items that makes it possible to draw conclusions concerning if respondents agree to each responsibility position. Note that some of the measures of positions are interlinked, it is for example possible to express support for equal burden sharing by choosing the option ‘equal share’ when asked about distribution of costs, instead of preferring to place the cost burden on the municipalities at the opposite location. Thus, although the survey questions are primarily used to draw conclusions about one particular responsibility position, we will also discuss implications for other positions, when applicable.

## Results

**Fig. 1:**
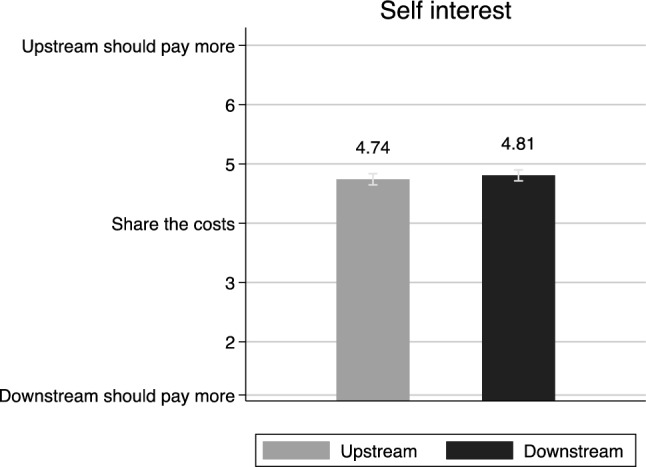
Politicians and Self-interest (mean). Note: The question asked was: How do you think that the costs for decreasing the risks that threatens the drinking water should be distributed between citizens in municipalities upstream (where water runs from) and citizens in municipalities downstream (where water runs to) the river? Seven-point scale was used to measure their attitudes, ranging from’citizens upstream should pay the full cost’ (1) to’citizens downstream should pay the full cost’ (7) with’equal share’ as a middle alternative (4). Confidence intervals at 95%

We start out with how the costs between upstream and downstream municipalities should be distributed. The first figure below refers to local politicians’ assessment on the distribution of the costs. The results in Fig. 1 reveal that politicians, both from upstream and downstream municipalities, are in agreement. Upstream communities should pay more than communities downstream (*M* = 4.74 (95% confidence interval [CI] = [4.64, 4.88]) versus *M* = 4.81 (95% CI [4.71, 4.90]), *p* = 0.32). Even though this is an experiment, self interest in this hypothetical setting is not the most important factor. Instead, politicians upstream are more willing to pay more to prevent risks. This finding challenges the idea of self-interest as always being the main problem. Our results show that politicians do think that polluters are more responsible, which means that self-interest does not necessarily need to be the main obstacle when politicians attempt to collaborate.

**Fig. 2:**
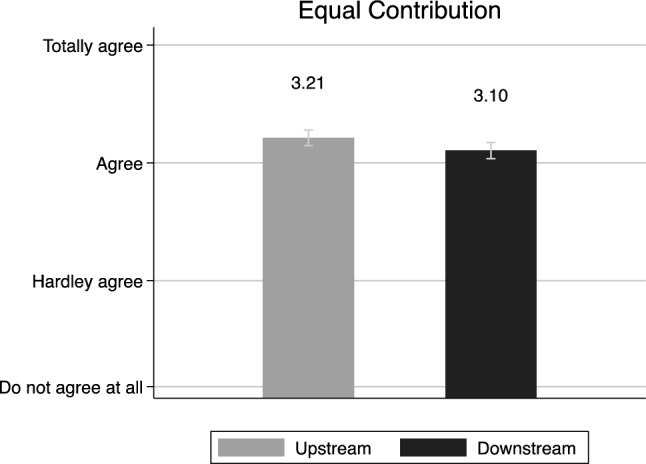
Politicians and Equal contribution (mean). Note: The statement was “Every single municipality is responsible for making investments in order to prevent events that pollutes the water, irrespective of the consequences do not affect the municipality’s citizens”. The respondents were asked to take a stand to by choosing from a scale with the following alternatives: totally agree, partly agree, hardly agree, do not agree at all. Confidence intervals at 95%

In the next step, we analyze the survey items relevant for a remedial responsibility position motivated by a preference for *equal contribution*. Figure 2 displays answers to the proposal that water fees should be raised in all municipalities to finance risk prevention measures. As agreement to this clearly is a stance that suggests a preference for equal contributions, the fact that both experimental groups display an overall agreement indicates that support for equal contribution can be verified. However, upstream respondents are slightly more positive than downstream respondents, which may be an indication of a position where they admit greater responsibility (*M* = 3.21 (95% confidence interval [CI] = [3.15, 3.28]) versus *M* = 3.10 (95% CI [3.04, 3.17]), *p* = 0.03). If events associated with water-quality risk only takes place in upstream locations, as presented in the scenario, an equal contribution would, in fact, be a downstream subvention of upstream risk prevention.

**Fig. 3:**
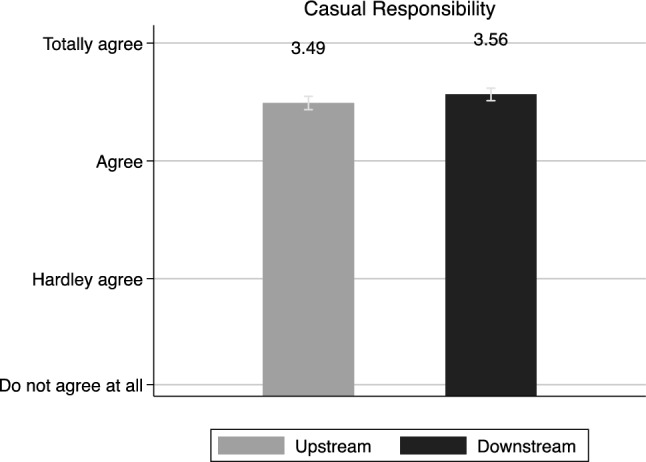
Politicians and Causal responsibility (mean). Note: The statement was “Municipalities upstream are responsible to make investments that reduces the risk for pollution of the water that can affect downstream municipalities”. The respondents were asked to take a stand to by choosing from a scale with the following alternatives: totally agree, partly agree, hardly agree, do not agree at all. Confidence intervals at 95%

Our third possible remedial responsibility position is motivated by a preference for *causal responsibility*. Here, given the scenario where the source of the pollution of the water is located upstream, both upstream and downstream actors should agree, in accordance with the Polluters Pays Principle, that municipalities in this location holds the responsibility for funding risk reduction or avoidance. Considering Fig. 3, this is also the case.

Downstream politicians are somewhat more positive to this suggestion than upstream respondents are. However, both groups agree with the statement, here interpreted as an overall support for upstream causal responsibility. As upstream politicians also agree to the proposal, they do not display self-interest but rather express a willingness to take on costs to prevent negative effects for downstream municipalities. Although downstream residents want to allocate a higher share of the costs to upstream municipalities (*M* = 3.49 (95% confidence interval [CI] = [3.43, 3.55]) versus *M* = 3.56 (95% CI [3.51, 3.62]), *p* = 0.07).

**Fig. 4:**
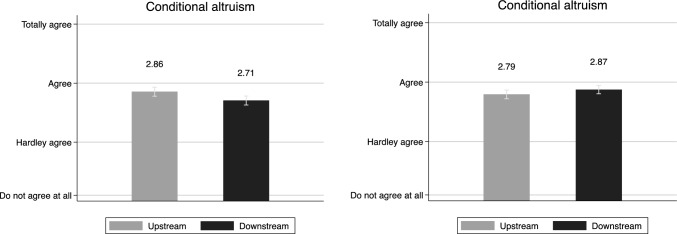
Politicians and Conditional altrusim (mean). Note: The statement for the figure to the left was “Municipalities downstream should contribute with money to municipalities upstream in order to help out with costs for measures that prevent pollution of the water”. The statement for the figure to the right was “If the water is polluted because of something occurring upstream, municipalities upstream should compensate downstream municipalities for the costs”. The respondents were asked to take a stand to by choosing from a scale with the following alternatives: totally agree, partly agree, hardly agree, do not agree at all. Confidence intervals at 95%

Lastly, we consider *conditional altruism*, that is, an agreement of an actor to take on costs only in the case that others do the same. Here, we use two items from the survey. As displayed in Fig. 4 (left figure), we ask respondents about their agreement to the proposal that municipalities located downstream should help municipalities upstream monetarily with costs associated with risk prevention. This item is slightly different than those associated with an *equal* burden sharing, as this only points out a certain contribution from downstream actors. The results suggest that neither upstream nor downstream politicians agree with the proposition that downstream municipalities should financially support their neighbors further up the river. Yet, upstream politicians are significantly more positive towards the idea (*M* = 2.86 (95% confidence interval [CI] = [2.78, 2.93]) versus *M* = 2.71 (95% CI [2.62, 2.78]), *p* = 0.01). Consequently, when asked about if upstream municipalities should compensate downstream municipalities (right figure) politicians do not approve. Both groups of politicians are skeptical, and there is no significant difference between them (*M* = 2.79 (95% confidence interval [CI] = [2.72, 2.87]) versus *M* = 2.87 (95% CI [2.80, 2.94]), *p* = 0.12). It seems like conditional altruism is something that politicians do not ascribe to.

## Discussion

Water systems are often common resources, flowing across administrative and political boundaries. The use and management of water systems therefore requires coordinated action among actors along the shoreline. For flowing water, a complication for coordination is upstream–downstream relations where what happens upstream affects downstream, but not the other way around. As water runs over boundaries, politicians need to choose whether to cooperate with other actors along the water systems, thus contributing to a sustainable water management for the collective, or to only consider the local self-interest.

In this article, we have examined politicians' perspectives on the allocation of responsibility for bearing the costs of preventing adverse events upstream. We have specifically focused on their positions as representatives of either upstream or downstream municipalities. If politicians are prepared to cooperate by taking on responsibility for preventing risks and managing incidents in a common water, the assumption is that it would facilitate the solution of the collective action problem associated with the coordination of actors located upstream and downstream.

We identified four theoretical responsibility positions. We used a scenario where an event in fact happened upstream, in order to trigger the politicians’ awareness of what could actually happen if preventive measures are not taken, and how that could affect downstream citizens.

Our results suggest that politicians, irrespective of location upstream or downstream, are inclined to support the idea that a polluter has a greater responsibility, in this case to take preventive action in order to not causing problems for other actors around the water system. First, both groups of politicians think that upstream actors should take on more of the costs associated with decreasing risks that threaten water quality. We interpret this as that upstream politicians are willing to accept a kind of preventive remedial responsibility, rather than trying to avoid all costs. Second, both groups support the suggestion that burdens should be shared equally, a result that confirms the upstream politicians’ willingness to take on costs in order to prevent problems downstream. It is, however, worth noticing that although the politicians agree with this suggestion, they do not on average ‘totally agree’, which means that they are not unanimously supportive. Third, even though downstream politicians significantly agree more, both groups of respondents think that upstream municipalities have causal responsibility in that they should be responsible for making investments to prevent problems downstream. Fourth, conditional altruism gains less support than the other suggestions, but the mean opinion is closer to ‘agree’ than ‘hardly agree’. Here, upstream politicians are slightly more positive to the suggestion that downstream municipalities should contribute to upstream cost for risk prevention.

These results challenge the idea of self-interest as the main obstacle to solving collective action problems with asymmetric payoffs and indicate that self-interest does not necessarily need to be the main obstacle when politicians attempt to collaborate. When given a scenario where an event upstream has negative effects downstream, decision makers allocated upstream display a willingness to accept causal responsibility, taking on remedial responsibility and act in (hydro)solidarity in order to prevent damages downstream. For downstream actors, support for equal burden sharing instead of insisting that upstream, as the causally responsible part, are fully responsible for preventive measures, may imply that they stand by principles such as that they as beneficiaries of upstream precautions should contribute (e.g. Page 2011). Even though the results are derived from an experiment, they do indicate that politicians in principle are open for taking responsibility even though it would not benefit them in a strict sense. A note of caution is that we cannot in this study rule out that the motivation behind the result signifies something else than a lack of self-interest. This is a question to explore in further research, where it would be interesting to reveal i the reasons behind politicians’ willingness to take on responsibility, through e.g. an interview study. Another issue to explore is if and in that case how politicians’ willingness to take on responsibility is aligned with public opinion and if a lack of congruence in this respect constitutes a problem for politicians’ willingness to take on remedial responsibility.

This study contributes to the understanding of the conditions for solving large-scale collective action problems (see Jagers et al 2019) in a particularly intriguing setting with asymmetric payoffs between the actors. Such settings are challenging as the actors do not cooperate on equal terms and differ in their incentives for engaging in collective action. Although upstream–downstream is an evident example of this kind of problem, also other settings can have the same character, e.g. when causes and effects are separated in time.

In addition, the study contributes to increase the knowledge on the conditions for successful collaboration on water resources. Previous research has identified several challenges and obstacles when it comes to actors’ willingness to engage in collaboration in order to achieve an effective way of dealing with water management problems (e.g. Bendz and Boholm 2019; Yoder et al. 2021). Our study implicates that watershed collaboration in an upstream–downstream setting could be facilitated or aggravated depending on how politicians understand the responsibility of their own and other municipalities and in relation to their location along the water. Thus, perceptions of how responsibility should be allocated between upstream and downstream could be one of the factors that affect how motivated local governments are to participate in collaborative governance (Hoornbeek et al 2016). This could be further explored in future research, including how responsibility perceptions interact with other factors apart form upstream/downstream location.

It is worth mentioning that Sweden is a country with a high level of trust in its institutions. In this context, Sweden can be considered a relatively "favorable" case for challenging self-interest with alternative norms (Rothstein and Stolle 2008). However, Sweden can also be viewed as a case where institutional design plays a significant role in fostering overall trust, thereby providing a critical mechanism for addressing collective action challenges (Martinangeli et al. 2023).

In the future, further research should be dedicated to understanding the conditions under which politicians are willing to engage in remedial actions, even in less favorable settings. If we can comprehend the circumstances in which politicians are inclined to prioritize the common good over their self-interest, we will make significant progress toward achieving sustainable water resource management.

### Supplementary Information

Below is the link to the electronic supplementary material.

Supplementary file1 (PDF 904 kb)

## Footnotes

1 Drinking water is regulated by the EU Drinking Water Directive Council Directive to ensure that water used for drinking is safe for human consumption. The Swedish National Food Agency is the regulatory authority on the national level responsible for drinking water quality. Its work is aligned with European rules and regulation for food safety by the European Food Safety Authority (EFSA). The 21 County Administration Boards are responsible for monitoring the implementation of the law locally. This includes environmental protection, natural resource management, water management and contingency planning.

2 The risk governance network for Göta Älv includes the County Administrative Board, the Swedish Transport Authority, the power producing company Vattenfall, Svenska Kraftnät (a government agency responsible for the national electricity grid), the Swedish Geotechnical Institute (SGI), municipalities, Göteborg Vatten (the city of Göteborg water works organization), the Vänern-Göta Älv River Council, the National Food Agency, the Swedish Agency for Marine and Water Management, the National Board for Building, Housing and Planning, the Swedish Civil Contingencies Agency, the Public Health Agency of Sweden and the Swedish Water and Wastewater Association (a stakeholder organisation set up by municipalities).

3 www.som.gu.se
