# Supplementary material for: A bridge over sustainable water: Politicians’ perceptions about the preconditions for collective action
Source: Ambio. 2024 Feb 7;53(5):764–75. doi: 10.1007/s13280-023-01975-5 (PMC10992074; doi:10.1007/s13280-023-01975-5)
Supplement: Supplementary file 1 — Supplementary file1 (PDF 904 kb) [file 13280_2023_1975_MOESM1_ESM.pdf]

Ambio

Supplementary Information

This supplementary information has not been peer reviewed.

Title: A bridge over sustainable water:

Politicians' perceptions about the preconditions for collective action

Authors: Anna Bendz, Patrik Öhberg

Anna Bendz
Associate Professor in Political Science (corresponding author)
Department of Political Science, University of Gothenburg,
Box 711, 405 30 Gothenburg.
E-mail: Anna.Bendz@pol.gu.se
Phone: +46 733 56 55 55

Patrik Öhberg
Associate Professor in Political Science
SOM-institute, University of Gothenburg
Seminariegatan 1B, 413 13 Gothenburg

Word count: 7175 (excluding references) 8438 (including references)

Figures and tables

Figure S1 Politicans and Self-interest, likert-scale plot (percent).

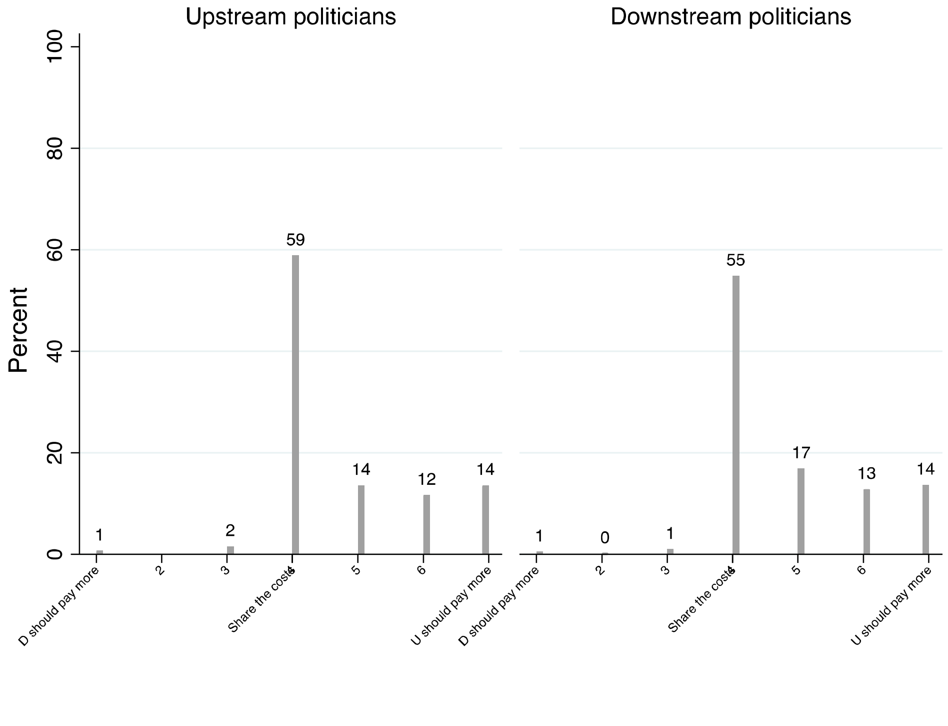

Note: The question asked was: How do you think that the costs for decreasing the risks that threatens the drinking water should be distributed between citizens in municipalities upstream (where water runs from) and citizens in

municipalities downstream (where water runs to) the river? Seven-point scale was used to measure their attitudes, ranging from ’citizens upstream should pay the full cost’ (1) to ’citizens downstream should pay the full cost’ (7) with ’equal share’ as a middle alternative (4).

Figure S2 Politicans and Equal contribution, likert-scale plot (percent)

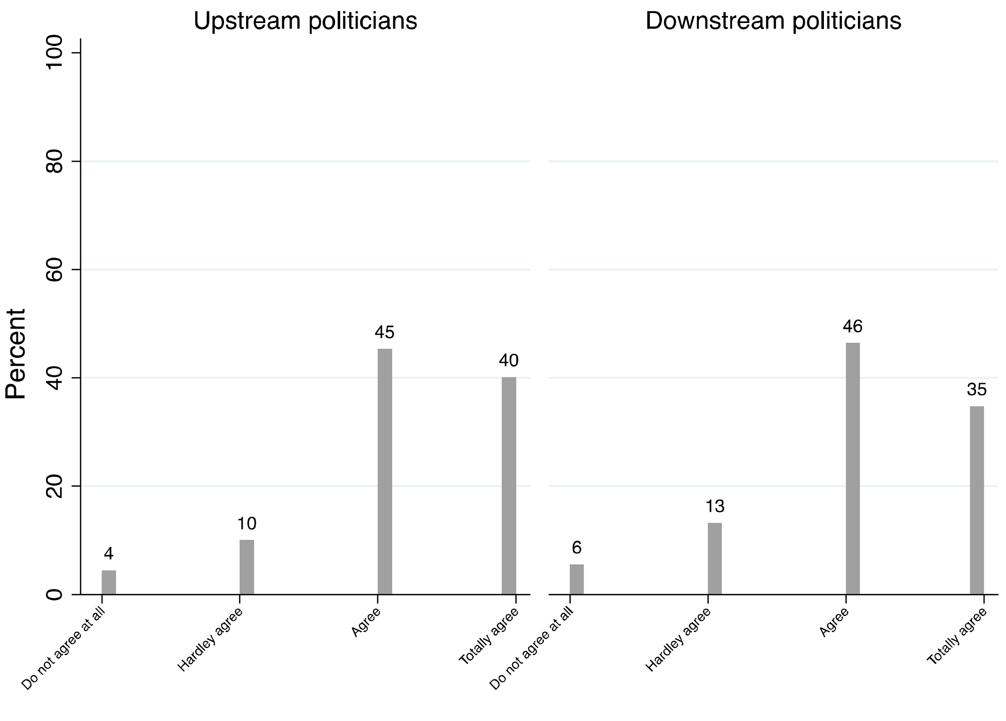

Note: The statement was “Every single municipality is responsible for making investments in order to prevent events that pollutes the water, irrespective of the consequences do not affect the municipality’s citizens”. The respondents were asked to take a stand to by choosing from a scale with the following alternatives: totally agree, partly agree, hardly agree, do not agree at all.

Figure S3 Politicans and Causal responsibility, likert-scale plot (percent)

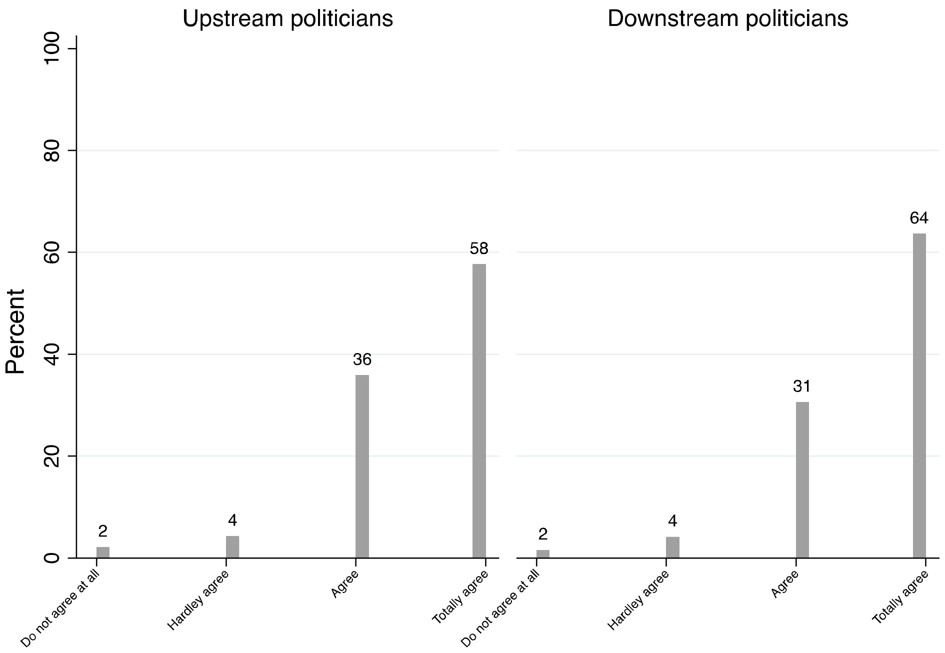

Note: The statement was "Municipalities upstream are responsible to make investments that reduces the risk for pollution of the water that can affect downstream municipalities". The respondents were asked to take a stand to by choosing from a scale with the following alternatives: totally agree, partly agree, hardly agree, do not agree at all.

Figure S4 Politicans and Conditional altrusim, likert-scale plot (percent)

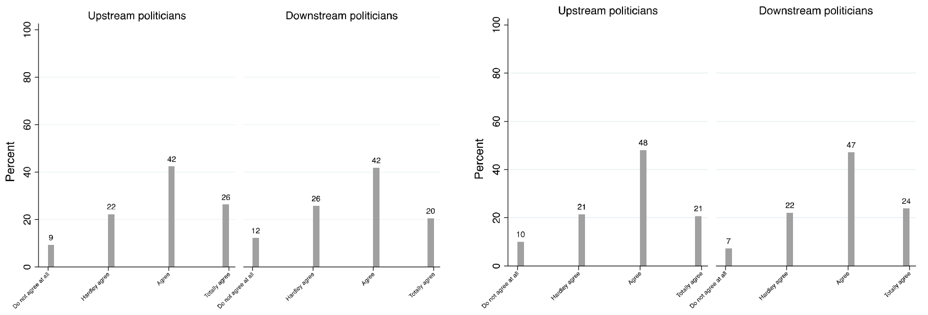

Note: The statement for the figure to the left was "Municipalities downstream should contribute with money to municipalities upstream in order to help out with costs for measures that prevent pollution of the water". The statement for the figure to the right was "If the water is polluted because of something occurring upstream, municipalities upstream should compensate downstream municipalities for the costs". The respondents were asked to take a stand to by choosing from a scale with the following alternatives: totally agree, partly agree, hardly agree, do not agree at all.

Table S1. Checks for randomization between experimental groups

	Mean scores (number of participants)				
**Control factors**	**Expgrp1**	**Expgrp2**	**Grand mean (n)**	**Sig.**	**F-quota**
Age	4.77<br>(590)	4.85<br>(610)	4.81<br>(1200)	0.25	1.30
Sex (women)	0.33<br>(587)	0.33<br>(605)	0.33<br>(1192)	0.86	0.03
Education at a University	0.66<br>(588)	0.65<br>(609)	0.66<br>(1197)	0.63	0.23
Ideology	2.93<br>(546)	2.85<br>(576)	2.89<br>(1122)	0.34	0.81
Total N	914	921			

Note: The age variable is separated into six categories (1 = under 30 years old, 2 = 30-39, 3 = 40-49, 4 = 50-59, 5 = 60-69, 6 = 70 or older. Education at a university is a dummy variable (0 = not studies at a university, 1 = studies at a university). Ideology contains of five categories (1 = clear to the left, 2 = slightly to the left, 3 = neither left nor right, 4 = slightly to the right, 5 = clear to the right).

Representativeness of the Panel of Politicians

To explore the representativeness of the Panel of Politicians, a study was conducted in October 2018 among respondents in the 2017 edition of Kommun- och Landstingsfullmäktigeundersökningen (KOLFU) (Andersson and Persson, 2018). KOLFU 2017 was sent out to all 12,829 local and regional politicians in late 2016 and included a question about whether the respondent was a member of or would like to join the Panel of Politicians. The respondents were given three alternatives: (1) Yes; (2) I am already a part of the Panel of Politicians; or (3) No thanks.[1] In the analysis, we merged alternative 1 and 2, thus creating a measurement for panel members which provided us an opportunity to examine whether panel members differed in a systematic way compared to the total sample of Swedish politicians at the sub-national level. Figures 1–3 below display the results from different sets of t-tests to detect skewness in the panel in terms of social characteristics and party membership. Significant differences ($p<0.001$) were found only in terms of gender and being a member of the Centre Party. Men and Centre Party members were found to be overrepresented in the panel, but all other characteristics were not.

Figure S5: T-tests, gender and place of origin

[1] 8,651 respondents answered this question, which constituted the sample.

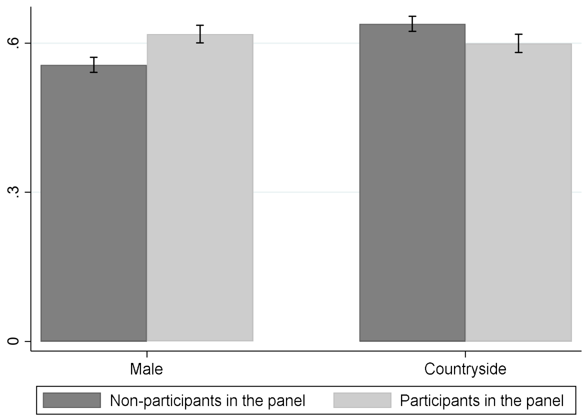

Comments: The figure is based on the means of two dummy variables: gender and place of origin. The gender variable was coded 0 = Female and 1 = Male. The place of origin variable was coded 0 = City and 1 = Countryside. Source: KOLFU 2017.

Figure S6 : T-tests, age

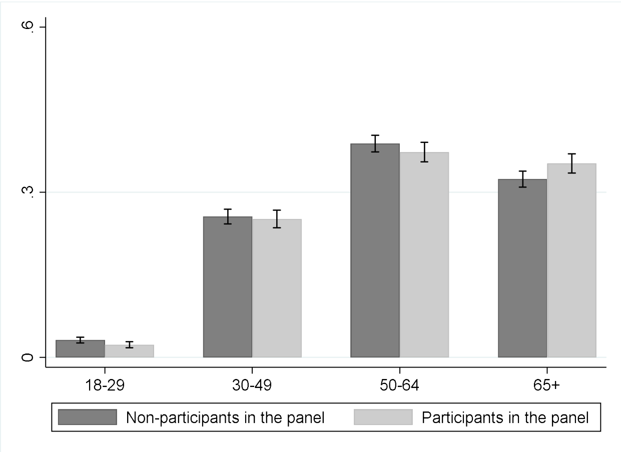

Comments: The age category in KOLFU 2017 was collected from register data from the Swedish Election Authority (Valmyndigheten). Based on the data, we constructed the four different age groups displayed in the figure. Source: KOLFU 2017

Figure S7: T-tests, party membership

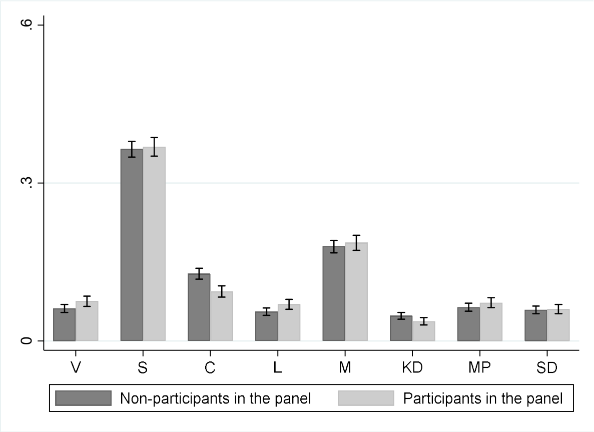

Comments: The figure is based on the means for the dummy variables for each party represented in the Swedish parliament (Riksdag). Source: KOLFU 2017
